# Association between depression and diabetes amongst adults in Bangladesh: a hospital based case–control study

**DOI:** 10.7189/jogh.05.020406

**Published:** 2015-12

**Authors:** Sheikh Mohammed Shariful Islam, Uta Ferrari, Jochen Seissler, Louis Niessen, Andreas Lechner

**Affiliations:** 1 Center for Control of Chronic Diseases (CCCD), International Center for Diarrhoeal Diseases Research, Bangladesh (ICDDR,B), Dhaka, Bangladesh; 2 Center for International Health (CIH), Ludwig–Maximilians Universität, Munich, Germany; 3 Cardiovascular Division, the George Institute for Global Health, Sydney, Australia; 4 Diabetes Research Group, Medical Department 4, Ludwig–Maximilians Universität, Munich, Germany; 5 Centre for Applied Health Research and Delivery, Liverpool School of Tropical Medicine, Liverpool, UK; 6 Clinical Cooperation Group Type 2 Diabetes, German Research Center for Environmental Health, Neuherberg, Germany; 7 Diabetes Research Group, German Center for Diabetes Research, Munich, Germany

## Abstract

**Methods:**

A matched case–control study was conducted among 591 consecutive patients with diabetes attending a tertiary hospital in Dhaka and 591 controls matched for age, sex and area of residence without diabetes not related with the index–case. Depression was measured using the Patient Health Questionnaire–9. Multivariate logistic regression was performed to examine the association between depression and diabetes.

**Results:**

The mean age (±standard deviation) of the participants was 50.4 ± 11.4 years, with a male to female ratio of 43:57. The prevalence of depression was 45.2% and 19.8% among cases and controls, respectively. In the multivariate analysis, mild as well as moderate to severe depression were significantly associated with diabetes and independent of sociodemographic factors and co–morbidity (adjusted odds ratio (OR) = 2.0, 95% confidence interval (CI) = 1.4–2.9 and adjusted OR = 6.4, 95% CI = 3.4–12.3; *P* < 0.001 for both).

**Conclusion:**

The high prevalence and strong association of depression in individuals with diabetes in Bangladesh suggests that depression should be routinely screened for patients with diabetes at the clinics and that management strategies adequate for resource–poor settings need to be developed. Further research to determine the pathophysiological role of depression in the development of diabetes is merited.

Diabetes and depression are two major non–communicable diseases which have become global epidemics and cause significant mortality and morbidity [1–3]. Worldwide, there are 382 million people with diabetes and within next two decades this number is projected to increase to 592 million with an increasing trend in the younger population [1]. The International Diabetes Federation (IDF) estimated that in 2013, diabetes caused 5.1 million deaths and cost US$ 548 billion in health care spending [1]. Diabetes is a major global cause of premature mortality, reduced quality of life, and imposes huge social and economic impact on health care systems, households and nations as a whole [1]. The global burden of disease study predicted that by 2030, depression is set to become the leading disease with 6.3% of the overall disease burden, and diabetes will be in 10th place with 2.3% of the overall disease burden as a percentage of the overall disability adjusted life years [4].

Diabetes and depression often present together and represent a major clinical challenge as the outcome of each condition can be worsened by the other [5]. Several studies have reported that comorbid diabetes and depression produced the greatest level of disability compared to other conditions, predicted sub–optimum outcomes, and incurred higher health care costs that increased with depression severity [6–9]. Despite high rates of comorbid depression in patients with diabetes, depression is often unrecognized and untreated in approximately two–thirds of patients in primary care settings [10].

The prevalence of both diabetes and depression are increasing in Southeast Asia [11]. Previous studies in Bangladesh have reported that the prevalence of depression among patients with diabetes was between 15.3–36% [12–15]. However, two of these studies had no control group [13–15] whereas the other two were population–based studies with relatively small numbers of incident cases of diabetes and insufficient data to examine sociodemographic and other factors potentially influencing the association of depression and diabetes [12,14]. Based on the data available, it is difficult to appreciate the true magnitude of the problem of depression among individuals with diabetes in Bangladesh as well as exclude important confounding factors. To close these knowledge gaps, we conducted a matched case–control study of individuals with and without diabetes at a large outpatient treatment facility in the Bangladeshi capital city, Dhaka. We hypothesized that persons with diabetes would have higher prevalence of depression than persons without diabetes.

## METHODS

### Study design, population and place

We conducted a matched case–control study among 1182 participants from January to July 2014 in the outpatient department (OPD) of the Bangladesh Institute of Health Sciences (BIHS) hospital. Detailed methods have been published elsewhere [16]. In brief, 591 consecutive patients with diabetes diagnosed by the BIHS attending physicians were recruited as cases. For each index–case, we recruited one control matched for age (±5 years), sex and area of residence from the persons accompanying other patients in the OPD waiting room. All individuals aged between 20–60 years were eligible for the study. Inclusion criteria for cases were: diagnosis of diabetes according to WHO criteria by attending BIHS physician. We excluded participants who were pregnant, had a terminal illness such as cancer or required urgent medical attention.

The BIHS is a 500–bed national–level tertiary health care covers all disciplines of medicine under a single roof having modern biomedical laboratory and research institute for diabetes affiliated with the Diabetes Association of Bangladesh and World Diabetic Federation. The OPD of BIHS hospital has one of the largest diabetes patient’s turnover in Bangladesh and serves a diverse population of about 2.2 million in Dhaka city and nearby districts.

### Data collection process

Data were collected by a team consisting of one project research physician, one research officer and three research assistants experienced in hospital data collection. The team was trained for 4 weeks on diabetes epidemiology, study design, study aims and objective, interview skills, research ethics, anthropometric and blood pressure measurements. The research tools and instruments were developed by the Health Economic Group of the International Diabetes Federation (IDF) and translated into Bengali as per WHO standards of translating and back–translating. The questionnaires were pre–tested in a similar setting in BIRDEM hospital OPD for 25 cases and 25 control subjects. Feedback from the field testing was used to improve the language and contents of the questionnaire and tools.

The questionnaire contained information about socio–demographic factors such as age, sex, marital status, education, occupation, income, history of depression, diabetes, family history of diabetes, smoking history, and self–reported complications (eye, hypertension, cardiovascular diseases, kidney diseases, etc). Weight, height, and hip and waist circumference were measured using standard protocol. Blood pressure was measured using digital blood pressure monitor (Omron, SEM–1, Omron Corporation, Japan). Two repeated measurements were recorded after an interval of 5 minutes, alternating right and left hands and the average of two readings was considered. Hypertension was defined as systolic blood pressure (SBP)≥140 mm Hg and/or diastolic blood pressure (DBP)≥90 mm Hg as per JNC 7 guideline. Blood tests on HbA1c were measured at the BIHS Research Laboratory.

Level of depression was measured using the Patient Health Questionnaire (PHQ–9) which consists of nine items on a 4–point Likert–type scale with scores ranging from 0–27 corresponding to the Diagnostic and Statistical Manual of Mental Disorder (DSM–IV) diagnostic criteria for major depressive disorder [17]. Depression scores of 0–4, 5–9, and ≥10 was used to classify minimal, mild and moderate to severe depression, respectively [18]. The PHQ–9 is one of the most widely used depression screening tools in primary health care and a cut–off score of ≥10 has shown to have 88% sensitivity and 88% specificity to diagnose major depression [19]. In this study we used a previously developed and evaluated Bengali version of PHQ–9. The PHQ–9 and its cut–off points have been validated in Bangladeshi population and considered to be reliable tool for diagnosis of depression [13].

### Data analysis

Data were entered into a Microsoft Access database with built–in range and consistency checks and analyzed using SPSS version 20 (IBM Corporation, NY, USA). Frequencies and percentages were calculated for categorical variables and mean±SD and median (Q1–Q3) were calculated for normality distributed and non–normally distributed continuous variable. T–test, χ^2^ and Mann-Whitney U tests were performed for differences between cases and controls. Univariate analysis was performed with diabetes as the dichotomous outcome variable. The category of the independent variable with the minimum level of association with diabetes was taken as reference value. Conditional logistic regression was performed to evaluate the association of depression and other independent variables with diabetes. Odds ratios (OR) are reported with their respective 95% confidence intervals (CI) and *P*–value. A *P*–value of less than 0.05 was considered significant.

## RESULTS

A total of 1265 participants were approached for this study and 1240 (98%) agreed to participate. Of those, 40 individuals were not included in the study (15 controls who had a history of diabetes, 8 cases who were pregnant, 17 cases who had no medical records available at the time of data collection). Another 18 participants were excluded before data analysis due to matching problems and incomplete information. The final sample therefore consisted of 1182 participants.

### Characteristics of the study participants

The study included 1182 participants with a male to female ratio of 43:57 and mean age (±standard deviation) of 50.4 ± 11.4 years. The majority of the participants were married and Muslims. About two–thirds of the participants completed secondary education or higher. About half of the participants were housewives, and one–third were service holders or businessmen. The overall median (Q1–Q3) household income was BDT 25 000 (15 000–60 000) or US$ 323.42 (194.05–776.20) and about two–thirds earned BDT 30 000 (US$ 388.10) or less per month (US$ 1 = BDT 77.3, 2014). Self–reported complications generally associated with diabetes (hypertension, cardiovascular diseases (CVD) and eye problems) were significantly higher among persons with diabetes than persons without diabetes (52.8% vs 19.3%, 10% vs 3.4% and 60.1% vs 38.1% respectively). Current tobacco use was higher among persons without diabetes than persons with diabetes (*P* = 0.04). The prevalence of hypertension measured by systolic blood pressure (SBP) and diastolic blood pressure (DBP) was higher for persons with diabetes than persons without diabetes (35.2% vs 28.1%, *P* = 0.009). Waist circumference and waist–hip ratio was significantly higher for persons with diabetes than persons without diabetes. Persons with diabetes also had a higher number of complications than persons without diabetes (1.76 ± 1.2 vs 2.05 ± 1.34). Persons with diabetes reported taking higher number of medication than persons without diabetes (3.67 ± 1.76 vs 1.79 ± 1.07) (**Table 1**).

**Table 1:** Characteristics of study participants

Variables	Case n = 591		Control n = 591		Total n = 1182		*P*–value
	n	%	n	%	n	%	
**Age (years):**							
Mean±SD	51.4 ± 11.6		49.5 ± 11.1		50.4 ± 11.4		0.004
<40	96	16.2	115	19.5	211	17.9	0.027
40–49	142	24.0	144	24.4	286	24.2	0.027
50–59	194	32.8	215	36.4	409	34.6	0.027
≥ 60	159	26.9	117	19.8	276	23.4	0.027
**Sex**							
Male	255	43.1	255	43.1	510	43.1	1.0
Female	336	56.9	336	56.9	672	56.9	1.0
**Marital status:**							
Married	476	80.5	517	87.5	993	84.0	0.001
Single	115	19.5	74	12.5	189	16.0	0.001
**Education:**							
No education	116	19.6	76	12.9	192	16.2	0.001
Primary	103	17.4	96	16.2	199	16.8	0.001
Secondary	190	32.1	178	30.1	368	31.1	0.001
Higher secondary and above	182	30.8	241	40.8	423	35.8	0.001
**Household monthly income (BDT):**							
Median (in thousands) (Q 1–3)	25 (15–42)		25 (16–40)		25 (15–60)		0.714
≤BDT 30 000	338	63.7	353	63.8	691	63.7	0.951
>BDT 30 000	193	36.3	200	36.2	393	36.3	0.951
**Occupation:**							
Unemployed	6	1.0	9	1.5	15	1.3	<0.001
Service	170	28.8	248	42.0	418	35.4	<0.001
Housewife	309	52.3	271	45.9	580	49.1	<0.001
Others (retired, labors, etc)	106	17.9	63	10.7	196	14.3	<0.001
**Self–reported complications:**							
Hypertension	312	52.8	114	19.3	426	36.0	<0.001
Cardiovascular diseases (CVD)	59	10	20	3.4	79	6.7	<0.001
Eye problems	355	60.1	225	38.1	580	49.1	<0.001
**Tobacco use:**							
Never	471	79.7	445	75.3	916	77.5	0.040
Former (stopped 6 months)	6	1.0	2	0.3	8	0.7	0.040
Current (in last 6 months)	114	19.3	144	24.4	258	21.8	0.040
**Depression (PHQ–9):**							
No or minimal depression (0–4)	324	54.8	474	80.2	798	67.5	<0.001
Mild depression (5–9)	167	28.3	100	16.9	267	22.6	<0.001
Moderate to severe depression (≥10)	100	16.9	17	2.9	117	9.9	<0.001
**Hypertension (SBP>140 /or DBP>90):**	208	35.2	166	28.1	374	31.6	0.009
**Body Mass Index (BMI):**							
Underweight (<18.5 kg/m^2^)	9	1.5	18	3.1	27	2.3	0.071
Normal (18.5 –24.9 kg/m^2^)	219	37.1	235	39.9	454	38.5	0.071
Overweight (25–29 kg/m^2^)	274	46.4	271	46.0	545	46.2	0.071
Obese (≥30 kg/m^2^)	88	14.9	65	11	153	13.0	0.071
**Waist circumference (WC):**							
Normal (≤90 cm M; ≤80 cm F)	154	26.1	215	36.4	369	31.2	<0.001
High (>90 cm M, >80 cm F)	437	73.9	376	63.6	813	68.8	<0.001
**Waist–to–hip ratio (WHR):**							
Normal (<0.90 M, <0.80 F)	29	4.9	55	9.3	84	7.1	0.003
High (≥0.90 M, ≥0.80 F)	562	95.1	536	90.7	1098	92.9	0.003
**Number of complications:**							
No complication	91	15.4	240	40.6	331	28.0	<0.001
1–3	451	76.3	328	55.5	779	65.9	<0.001
>3	49	8.3	23	3.9	72	6.1	<0.001
Median (IQR)	2 (2)		2 (2)		2 (2)		<0.001
**Number of medications:**							
1–2	157	27.2	98	80.3	255	36.4	<0.001
3–4	259	44.8	21	17.2	280	40.0	<0.001
>4	162	28.0	3	2.5	165	23.6	<0.001
Median (IQR)	3(3)		1(1)		3(2)		<0.001

SD – standard deviation, IQR – interquartile range, Q – quartile, BDT – Bangladeshi Taka, PHQ – Patient Health Questionnaire, SBP – systolic blood pressure, DBP – diastolic blood pressure, M – male, F – female, BMI – body mass index (kg/m^2^)

### Prevalence of depression

**Figure 1:**
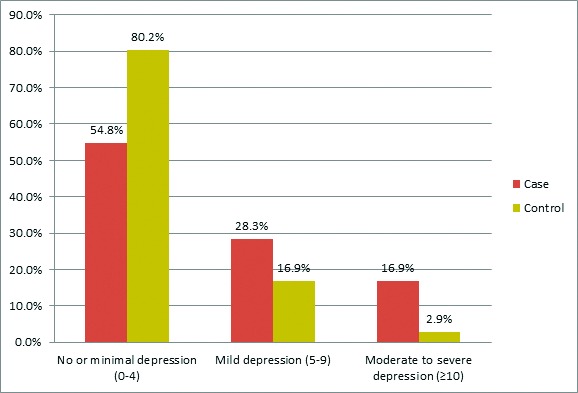
Prevalence of depression among study participants using PHQ–9.

The prevalence of depressive illness was found higher among persons with diabetes (28.3%) than persons without diabetes (16.9%; *P* < 0.001). The prevalence of moderate to severe depression was 16.9% in persons with diabetes vs 2.9% in persons without diabetes (*P* < 0.001) (**Table 1** and **Figure 1**).

### Association between diabetes and depression

**Table 2** shows the univariate analysis of factors associated with diabetes with unadjusted OR and 95% CI. No depression or minimal and moderate to severe depression were significantly associated with diabetes (OR = 2.7, 95% CI = 2.0–3.8) and (OR = 9.9, 95% CI = 5.4–18.0), respectively. Other factors found to be significantly associated with diabetes were age ≥40 years, secondary and higher education (inverse association), housewife or other occupation (such as retirees, day laborers), marital status single, obesity, hypertension and having higher number of complications (**Table 2**).

**Table 2:** Univariate analysis of factors associated with diabetes

Variables	Odds ratio (OR)	Confidence interval	*P*–value
**Depression:**			
Minimal depression (0–4)	Ref		
Mild depression (5–9)	2.7	2.0–3.8	<0.001
Moderate to severe depression (≥10)	9.9	5.4–18.0	<0.001
**Education:**			
No education	Ref		
Primary	0.7	0.5–1.1	0.093
Secondary	0.7	0.5–1.0	0.034
Higher secondary and above	0.5	0.3–0.7	<0.001
**Age:**			
<40	Ref		
≥40	4.2	1.7–10.2	0.002
**Household monthly income (BDT):**			
≤BDT 30 000	Ref		
>BDT 30 000	1.0	0.8–1.3	0.842
**Occupation:**			
Unemployed	Ref		
Service	1.1	0.4–3.2	0.876
Housewife	3.1	1.0–9.6	0.048
Others (retired, labors, etc)	3.2	1.0–9.7	0.046
**Marital status:**			
Married	Ref		
Single	1.7	1.2–2.4	0.001
**BMI:**			
Underweight (<18.5 kg/m^2^)	0.5	0.2–1.2	0.098
Normal (18.5–24.9 kg/m^2^)	Ref		
Overweight (25–29.9 kg/m^2^)	1.1	0.9–1.4	0.435
Obese (≥30 kg/m^2^)	1.5	1.0–2.1	0.044
**Hypertension:**			
Absent	Ref		
Present	1.4	1.1–1.8	0.009
**Number of complications:**			
No complication	Ref		
1–3	4.0	2.9–5.6	<0.001
>3	6.7	3.7–12.0	<0.001

BMI – body mass index (kg/m^2^)

**Table 3** presents the results of conditional logistic regression analyses for factors associated with diabetes after adjusting for confounders. No depression or minimal and moderate to severe depression were significantly associated with diabetes (OR = 2.0, 95% CI = 1.4–2.9) and (OR = 6.4, 95% CI = 3.4–12.3) respectively. Having 1–3 complications (OR = 3.1, 95% CI = 2.2–4.4), ≥3 complications (OR = 3.1, 95% CI = 1.5–6.2), other occupations (OR = 4.9, 95% CI = 1.3–18.0) and completing higher secondary education and above (OR = 0.52, 95% CI = 0.3–0.8) were also significantly associated with diabetes, controlling for other confounding variables.

**Table 3:** Conditional logistic regression analyses for factors associated with diabetes

Variables	Odds ratio (OR)	Confidence interval	*P*-value
**Depression:**			
No or minimal depression (0–4)	Ref		
Mild depression (5–9)	2.0	1.4–2.90	<0.001
Moderate to severe depression (≥10)	6.4	3.4–12.3	<0.001
**Age (years):**			
<40	Ref		
≥40	1.71	0.65–4.47	0.278
**Education:**			
No education	Ref		
Primary	0.76	0.46–1.24	0.269
Secondary	0.76	0.48–1.19	0.224
Higher secondary and above	0.52	0.33–0.83	0.006
**Occupation:**			
Unemployed	Ref		
Service or business	1.70	0.49–5.83	0.400
Housewife	3.67	0.98–13.75	0.054
Others	4.92	1.34–18.00	0.016
**Marital status:**			
Married	Ref		
Single	1.50	1.00–2.26	0.051
**BMI:**			
Underweight (<18.5 kg/m^2^)	0.32	0.11–0.96	0.042
Normal (18.5–24.9 kg/m^2^)	Ref		
Overweight (25 –29.9 kg/m^2^)	1.14	0.83–1.55	0.420
Obese (≥30 kg/m^2^)	1.36	0.85–2.17	0.194
**Hypertension:**			
Absent	Ref		
Present	1.24	0.91–1.68	0.171
**Number of complications:**			
No complication	Ref		
1–3	3.07	2.15–4.38	<0.001
>3	3.06	1.52–6.17	0.002

BMI – body mass index (kg/m^2^)

## DISCUSSION

This study, to the best of our knowledge, is the first matched case–control study determining the prevalence of depression among people with and without diabetes in Bangladesh that also measures the association between depression and diabetes. Our study showed that depression, particularly in a moderate to severe form, is much more common among those with diabetes than those without the disease. In addition, we found that the association of depression and diabetes is independent of socio–demographic factors and diabetes–associated complications.

Several longitudinal studies have reported that increased depressive symptoms at baseline are associated with incident type 2 diabetes [7,20,21]. Several factors associated with depression, such as physical inactivity, hypercaloric diet, neuroendocrine and inflammatory responses resulting in increased cortisol, catecholamines, and cytokines can induce insulin resistance leading to the development of diabetes [7]. A meta–analysis showed that the risk of developing type 2 diabetes was 37% higher in depressed adults than in non–depressed adults [22].

Conversely, the psychosocial demands of diabetes management, lifestyle change, incidence of complications and resulting functional impairment may influence depression severity, decrease quality of life, and contribute to prolonged or recurrent episodes of depression [23]. Depression in patients with chronic illness might cause nonspecific amplification of physical symptoms associated with the medical condition [24]. Compared to non–depressed patients, patients with major depressive disorders were 2 to 5 times more likely to report the presence of 10 diabetic symptoms after controlling for a number of diabetes complications [25]. These results are in line with our findings of an increased number of complications associated with diabetes which might lead to or aggravate depression.

Depressive symptoms are associated with decreased glycemic control and increased diabetic complications, which worsen depression and lessen response to antidepressant treatment [26]. Previous studies have shown that the correlation between depression and poor diabetic self–care is consistent across diverse socioeconomic and cultural groups [27,28]. Comorbid depression in patients with diabetes is also associated with increased numbers and severity of diabetic symptoms and complications [29,30]. A meta–analysis demonstrated a clinically significant relation between depression and several diabetic complications [31]. Our results show that patients with more complications had 3 times the odds to be significantly associated with diabetes.

Previous studies have shown that type 2 diabetes is associated with an increased risk of depressive symptoms [32,33]. A Bangladeshi study reported 31.6% of comorbid depression among patients with type 2 diabetes while the prevalence of depression in persons without diabetes was 12.6%, which is similar to our findings [14]. Another study in Bangladesh reported 36.2% of participants with moderate to severe depression, which was significantly higher among females [34]. A worldwide survey by WHO reported that 9.3% of patients with diabetes also had depression [6]. A meta–analysis reported that people with type 2 diabetes have a 24% increased risk of incident depression compared with people without diabetes [35]. A study in China reported that depression was three times higher among persons with diabetes compared to those without diabetes [36]. Our results show a much higher prevalence of depressive symptoms among patients with diabetes compared to previous studies in Bangladesh, which might be due to selection of samples from a specialized hospital as well as the use of different scale and cut–off values to measure depression. Also, participants with moderate to severe depression in our study had 6.4 times higher odds of having diabetes, which is almost double what is reported by a study from China [36].

A recent systematic review reported that the prevalence of depression among individuals with diabetes is higher in population with low socioeconomic status in low–and–middle–income countries. However, the available evidence base was small [10]. We found that the association of diabetes and depression was independent of an individual’s education and household income in our sample. Additionally, it was not affected by other socio–demographic factors, BMI, hypertension, or the number of diabetes–associated complications.

Even in well–funded health care systems, depression is under diagnosed and undertreated in individuals with diabetes [5]. In Bangladesh, where there is a shortage of trained workforce in mental health and diabetes, patients with comorbid diabetes and depression are even less likely to receive adequate management for both conditions [37]. This may contribute to the fact that diabetes management in Bangladesh is suboptimal even in the best clinical settings, and the majority of the patients present with high rates of complications [38].

### Strengths and limitations

The strength of this study is the matched case–control design which controlled for the age, sex and area of residence of the study participants during the recruitment stage. Both case and controls were recruited at the same time, under similar conditions, by the same research assistants and from the same source population reducing confounding bias. The limitations of this study include that controls were selected on the basis of self–reported absence of diabetes, which could not be verified by laboratory investigations. However, our study physician ensured that the controls were not on any anti–diabetic medications. We used PHQ–9 which was not designed to measure clinical depression. However, PHQ–9 is an efficient and valid tool and has been commonly used to identify depression in primary health care in previous studies [13,39]. Furthermore, we measured depression at a single–time point and did not consider the use of antidepressants, which might have misclassified our participants. Finally, our data on complications are self–reported by participants for cardiovascular diseases, eye problems and kidney diseases which could not be verified by clinical or laboratory investigations. They were however verified to the extent possible by a review of the participants’ medical records. Well–designed longitudinal studies with objective measurements of clinical complications and measures of neuroendocrine markers will help to establish the direction of association and pathophysiology of both depression and diabetes among the Bangladeshi population.

## CONCLUSION

The prevalence of depression, particularly moderate to severe, is very high among adult Bangladeshis with diabetes. Therefore, patients with diabetes should be routinely screened for depression in Bangladesh and probably similar other developing countries. Management strategies and guidelines adequate for the country level need to be developed and further research to determine the pathophysiological role of depression in the development of diabetes in Southeast Asians is merited.
